# Electrical Performance of Polymer-Insulated Rail Brackets of DC Transit Subjected to Lightning Induced Overvoltage

**DOI:** 10.3390/ma14071684

**Published:** 2021-03-29

**Authors:** Farah Asyikin Abd Rahman, Mohd Zainal Abidin Ab Kadir, Ungku Anisa Ungku Amirulddin, Miszaina Osman

**Affiliations:** 1 Institute of Power Engineering (IPE), Universiti Tenaga Nasional (UNITEN), Kajang 43000, Selangor, Malaysia; anisa@uniten.edu.my (U.A.U.A.); miszaina@uniten.edu.my (M.O.); 2 Centre for Electromagnetic and Lightning Protection Research (CELP), Advanced Lightning, Power and Energy Research Centre (ALPER), Universiti Putra Malaysia (UPM), Serdang 43400, Selangor, Malaysia; mzk@upm.edu.my

**Keywords:** DC transit, EMTP-RV, fourth rail, indirect lightning, lightning induced overvoltage, surge arrester

## Abstract

The fourth rail transit is an interesting topic to be shared and accessed by the community within that area of expertise. Several ongoing works are currently being conducted especially in the aspects of system technical performances including the rail bracket component and the sensitivity analyses on the various rail designs. Furthermore, the lightning surge study on railway electrification is significant due to the fact that only a handful of publications are available in this regard, especially on the fourth rail transit. For this reason, this paper presents a study on the electrical performance of a fourth rail Direct Current (DC) urban transit affected by an indirect lightning strike. The indirect lightning strike was modelled by means of the Rusck model and the sum of two Heidler functions. The simulations were carried out using the EMTP-RV software which included the performance comparison of polymer-insulated rail brackets, namely the Cast Epoxy (CE), the Cycloaliphatic Epoxy A (CEA), and the Glass Reinforced Plastic (GRP) together with the station arresters when subjected by 30 kA (5/80 µs) and 90 kA (9/200 µs) lightning currents. The results obtained demonstrated that the GRP material has been able to slightly lower its induced overvoltage as compared to other materials, especially for the case of 90 kA (9/200 µs), and thus serves better coordination with the station arresters. This improvement has also reflected on the recorded residual voltage and energy absorption capacity of the arrester, respectively.

## 1. Introduction

A fourth rail traction technology came into the picture as a post development of the third rail traction technology. The main advantage of the fourth rail traction is that the running rails would not have to carry either polarity of current, thus avoiding the stray current issue which can lead to corrosion and even arcing, especially if the tunnel segments are not electrically bonded [1]. For instance, in the case of London Underground, which runs through Victorian mains that predated the railway section, where the pipe segments were never constructed to serve that purpose [2]. London Underground is one of the three urban transits in the world that employs the fourth rail traction technology. The other two are Milan Metro Line 1 and the Light Rail Transit (LRT) Kelana Jaya Line in Italy and Malaysia, respectively [1,2]. The LRT Kelana Jaya line is distinguished by not only running underground but being the only one that partially runs on the elevated track. This track is 42.1 km long and 17 m above the ground. Whilst the underground route is 4.3 km stretching as far as five consecutive stations running underneath the Kuala Lumpur Central Business District en route to Gombak from Putra Heights [3]. However, running on the elevated track has given the stress on the operation of direct current (DC) urban transit and its maintenance credibility, i.e., lightning protection, especially when the track is built and operated in a city that is ranked at the fifth-highest lightning activity in the world [4].

The LRT Kelana Jaya line has been in operation for the last 21 years and throughout its two decades of service, the line has experienced a number of technical disturbances and traction power loss due to lightning. The first incident was reported on 15 November 2010 when the line was struck by lightning and caused the train to fail to stop at its four out of five subway stations namely Kuala Lumpur City Centre (KLCC), Kampung Baru, Dang Wangi, and Masjid Jamek [5]. Three years later, another lightning strike has caused the traction power loss in some areas, which consequently caused delays and left the commuters stranded at the stations [6]. The probable cause of these incidents was reported due to the health and insulation strength of the electrical equipment.

Typically, an overhead line running transit runs in an open area and thus, is highly susceptible to lightning. Hence, the overwhelming lightning protection studies and research development were dedicated to this type of transit. This is in contrast to the third rail transit studies which are normally underground and thus, protected from the lightning. In this case, the issue seemed to be highly attentive to the stray current. On another note, if the third rail system runs on the elevated track, that particular section may be shielded by neighbouring buildings [7]. However, this study focuses on an elevated fourth rail transit which consists of civil and electrical structures that is distinguished from other systems. With the limitation of the available references, this study intends to fill in the research gap pertinent to the lightning surge analyses of an urban transit on the fourth rail traction, with particular interest in the insulated rail bracket and station lightning arrester. 

## 2. Case Study and Methods

### 2.1. The Kelana Jaya Line

Boasted as the busiest light rail transit in Malaysia with 46.4 km line length, the LRT Kelana Jaya line runs seamlessly between the north eastern suburbs of Kuala Lumpur to Petaling Jaya and west of Kuala Lumpur with 37 stations [1]. This electric transit train runs on a 750 VDC fourth rail system and has been operating for two decades. The LRT Kelana Jaya line is unique in its own way due to its elevated fourth rail traction and distinct power rail arrangement, as compared to the ones in Milan and London. The power rails of the LRT Kelana Jaya line are mounted parallel with each other, with an insulator in place to isolate the positive rail (third rail) and the negative rail (fourth rail). This insulator is known as the insulated rail bracket, which is the primary subject for this study. 

In this study, a section of Setiawangsa Station to Damai Station was chosen due to its elevated routes which are located between Sri Rampai Station and Ampang Park Station. To the best of the author’s knowledge, there is no surge arrester installed along the 3 km power rails, which are considered in this study. Generally, the arresters are installed in the vicinity of the electrical equipment to be protected, for the purpose of minimizing the risk of equipment failure due to high surges. 

### 2.2. Power Rails

**Figure 1:**
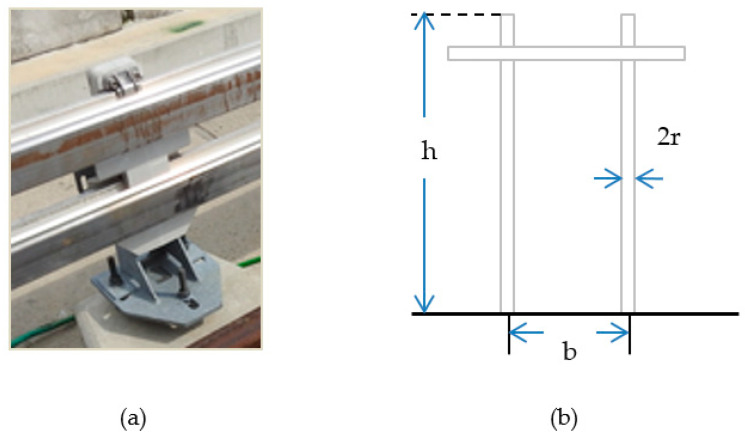
(**a**) The orientation of the power rails. (**b**) The H-frame tower model.

The limited references of the fourth rail transient study has provided the idea of conceptualization of the tower surge impedance model to portray the power rail representation. These two structures are more or less similar in terms of the conductor physical structure. Although the power rails serve the same purpose as any transmission line, they do not share the same conductor structure. Unlike transmission lines whose cross sections are circular and constructed from more than one conductor wire, power rails are made up from a solid conductor with an irregular cross section [8], and therefore sharing more of a resemblance with the structure of a transmission tower. Furthermore, even though the lightning response of a transmission tower is an electromagnetic phenomenon, yet researchers and physicists are able to represent the tower structure by means of line sections and circuit elements (e.g., resistors, inductors, and capacitors) [9]. This makes the power rail impedance adaptation of the tower surge impedance easier to be simulated in the Electromagnetic Transients Program—Restructured Version (EMTP-RV) software. There are many styles and perspectives concerning the tower surge impedance. However, the following Equations (1)–(3) [9], appear to be more appropriate for representing the rail configuration (Figure 1a) with the equivalent shape of transmission line tower (Figure 1b) [9], known as the H-frame tower. As for the ground, the cemented guideway demonstrates a perfect conducting ground [10].

(1) $$Z_{t}\;=\;\frac{Z_{s}+Z_{m}}{2}$$

With

Z_s_ = 60ln(L/r) + 90(r/L) − 60 (2)

Z_m_ = 60ln(L/b) + 90(b/L) − 60 (3)

where L is the length of the power rail (m), r is the radius of the power rail (m), b is the distance between the rails (m), Z_s_ is self-impedance (Ω), and Z_m_ is mutual impedance (Ω).

### 2.3. Insulated Rail Bracket

The LRT Kelana Jaya line has a distinct power rail arrangement, i.e., mounted parallel to each other. For this reason, an insulator is essential to isolate the positive rail (third rail) from the negative rail (fourth rail), on top of acting as the rail support. The conceptualization of the respective power rail insulated rail brackets is realized in the same manner as per the power line insulator which embodies a capacitor, apart from different dielectric insulations to be used to represent the Cast Epoxy (CE), the Cycloaliphatic Epoxy A (CEA), and the Glass Reinforced Plastic (GRP), with the CE bracket set as the LRT line rail bracket. Equation (4) quantifies an appropriate adaptation of capacitance to be used in this study.

(4) $$C=\frac{{A\varepsilon }_{0}\varepsilon_{r}}{d}$$

where C is capacitance, A is the area between the plates (m^2^), 0.006764 m^2^, d is the distance between the plates (m), 0.083 m, ε_0_ is the permittivity of free space, 8.85 × 10^−12^ F/m, ε_r_ is the permittivity of GRP dielectric, 5 [11], ε_r_ is the permittivity of CE dielectric, 4 [12], and ε_r_ is the permittivity of CEA dielectric, 3.5 [13]. 

In the presence of high-voltage stress on the power rails, where the structures are separated only by air and insulated rail brackets every 5 m, the flashover is always expected to occur across the insulated rail brackets, unless surge arresters are installed along the power rails. This is due to the fact that as the stress increases to a point that exceeds the electric strength of the air (3 × 10^6^ V/m) [14,15], and the bracket material, a spark travels from one conductor rail to the other and if the stress is sustained, this may also be followed by a continuous arc, hence, a flashover. In modelling this occurrence, a flashover switch was connected across the insulated rail brackets that closed accordingly when the presence of the high-voltage stress exceeds the calculated flashover voltage (FOV) of the bracket material. The bracket FOV was calculated by Equation (5). 

The flashover voltage of the insulated rail bracket was calculated through this calculation [16,17]:

(5) $$V=400+\left(\frac{710}{t^{0.75}}\right)\times \ell$$

where V is the flashover voltage (kV), t is the time to flashover (range 0.5 to 16 µs) and in this study it is 2 µs [17], and ℓ is the insulated rail brackets length (m), 0.076 m. According to IEEE STD 1410-2010, to obtain an estimated value for the critical flashover (CFO) for wet conditions, the dry CFO values were multiplied by 0.8 ± 0.1 [18].

On the other hand, the IEEE Standard 1410 defines the basic insulation level (BIL) as the crest value of a standard lightning impulse, in which the insulation exhibits a 90% probability of withstands (or a 10% probability of failures) under specified conditions [18] and can be expressed as in Equation (6) [19,20]:

(6) $$\mathrm{BIL}=\mathrm{CFO}\;\left(1-1.28\;\frac{\sigma_{f}}{\mathrm{CFO}}\right)$$

with σ_f_ as the coefficient of the variation, known as sigma. For lightning, the sigma is between 2% to 3%. 

### 2.4. Traction Substation Surge Arrester 

**Figure 2:**
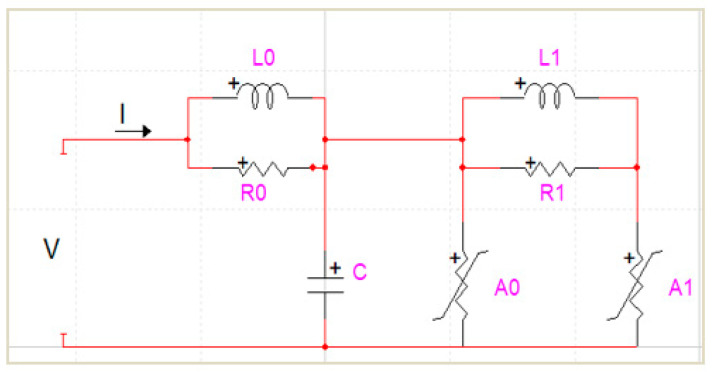
The Institute of Electrical and Electronics Engineers (IEEE)model as the surge arrester representation [24].

The location of the arresters for this study were assumed to be at the entrance of the DC side of the traction substation. The arrester was modelled based on the Institute of Electrical and Electronics Engineers (IEEE) model where it takes into account both the electrical data (residual voltages), and the physical parameters (overall height, block diameter, number of columns) [14], as well as it works splendidly with the impulse current flow with a wave front between 0.5 to 45 μs [21,22]. The respective designed IEEE model is shown in Figure 2 with its parameters established through Equations (7)–(11):

(7) $$L_{1}=\;\frac{15d}{n}\;\mu H$$

(8) $$R_{1}=\frac{65d}{n}\;\Omega$$

(9) $$L_{0}=\;\frac{0.2d}{n}\;\mu H$$

(10) $$R_{0}=\;\frac{100d}{n}\;\Omega$$

(11) $$C\;=\;\frac{100n}{d}\;\mathrm{pF}$$

where d is the estimated height of the arrester (m) and n is the number of arrester columns in parallel [23]. 

### 2.5. Indirect Lightning and LIOV Modelling

Indirect lightning occurs when a strike from lightning hits the ground or an object is in the vicinity of the DC transit track. This event subsequently initiates a travelling transient voltage via conductors and inductively induces a few hundred of kilovolts that can damage or destroy any unprotected electronic components [25]. The indirect lightning was modelled using the Heidler channel base current model or known as the Heidler function. Whilst the lightning induced overvoltage (LIOV) was modelled based on the Rusck model. 

The proposed Heidler function is shown in the following Equations (12) and (13) [26]:

(12) $$I\left(0,t\right)=\frac{I_{0}}{\eta }\times \frac{{\left(\frac{t}{\tau_{1}}\right)}^{n}}{1+{\left(\frac{t}{\tau_{1}}\right)}^{n}}\;\times {\;e}^{\left(-\frac{t}{\tau_{2}}\right)}$$

where

(13) $$\eta \;=\;\exp\;[-(\frac{\tau_{1}}{\tau_{2}})\;\times {\left(\frac{{n\tau }_{2}}{\tau_{1}}\right)}^{\left(1/n\right)}]$$

I_0_ is the channel base current peak value of the Heidler function, τ_1_ is the time constant of the wave front of the Heidler function, τ_2_ is the time constant of the wave decay of the Heidler function, n is the exponent of the Heidler function (usually holds a value between 2 and 10), and ***η*** is the amplitude correction factor of the Heidler function. 

In reproducing the lightning current waveshape with a concave rising portion, Equation (12) was repeated twice and resulted in Equations (14) and (15) [27,28,29]:

(14) $$I\left(0,t\right)=\frac{I_{1}}{\eta_{1}}\times \frac{{\left(\frac{t}{\tau_{11}}\right)}^{n_{1}}}{1+{\left(\frac{t}{\tau_{11}}\right)}^{n_{1}}}\;\times {\;e}^{\left(-\frac{t}{\tau_{12}}\right)}+\;\frac{I_{2}}{\eta_{2}}\times \frac{{\left(\frac{t}{\tau_{21}}\right)}^{n_{2}}}{1+{\left(\frac{t}{\tau_{21}}\right)}^{n_{2}}}\times {\;e}^{\left(-\frac{t}{\tau_{22}}\right)}$$

where

(15) $$\eta_{1}\;=\;\exp\;[-(\frac{\tau_{11}}{\tau_{12}}\;)\;\times {\left(\frac{n_{1}\tau_{12}}{\tau_{11}}\;\right)}^{\left(1/n_{1}\right)}]\;\mathrm{and}\;\eta_{2}\;=\;\exp\;[-(\frac{\tau_{21}}{\tau_{22}}\;)\;\times {\left(\frac{n_{2}\tau_{22}}{\tau_{21}}\;\right)}^{\left(1/n_{2}\right)}]$$

While the Rusck model can be obtained from Equation (16):

(16) $$v\;\left(x,t\right)=\frac{\zeta_{0}I_{0}h}{4\pi }\beta \left(\frac{\mathrm{ct}-x}{d^{2}+\beta^{2}{\left(\mathrm{ct}-x\right)}^{2}}\;\left(1+\frac{x+\beta^{2}\left(\mathrm{ct}-x\right)}{\sqrt{{\left(\beta \mathrm{ct}\right)}^{2}+\frac{x^{2}+d^{2}}{\gamma^{2}}}}\right)+\;\frac{\mathrm{ct}+x}{d^{2}+\beta^{2}{\left(\mathrm{ct}+x\right)}^{2}}\left(1+\frac{x+\beta^{2}\left(\mathrm{ct}+x\right)}{\sqrt{{\left(\beta \mathrm{ct}\right)}^{2}+\frac{x^{2}+d^{2}}{\gamma^{2}}}}\right)\right)\;$$

where $\beta \;\mathrm{is}\;\mathrm{the}\frac{v}{c}$ (v is the return stroke velocity and c is the speed of light), ζ0 represents 376.730313 Ω (free space characteristic impedance), I_0_ is the channel base current peak, d is the horizontal distance, x is the vertical distance from a lightning stroke, h is the guideway height, and γ is $1/\sqrt{1-\beta^{2}}$.

### 2.6. EMTP-RV Modelling

**Figure 3:**
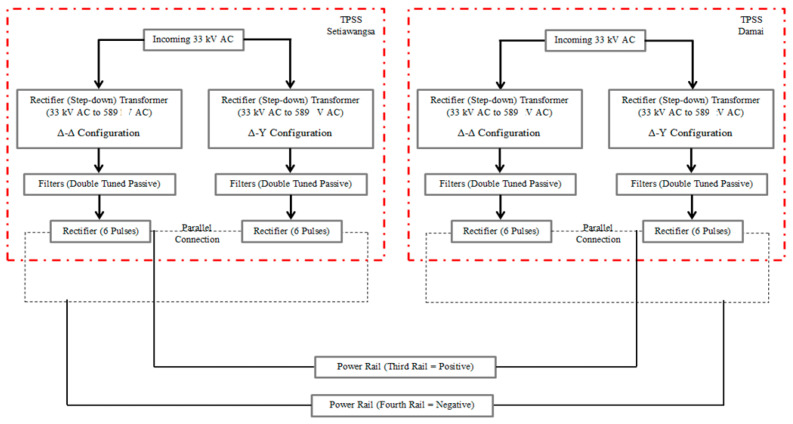
Block diagram of the LRT Kelana Jaya system.

As highlighted in Figure 3, a complete system was modelled as per the Single Line Drawing (SLD) provided. Any feature of the LRT line power system that exhibits a low or zero reaction towards the intended study was omitted in order to simplify the model and to not be a hindrance in terms of the computational speed. The salient features such as transformers, rectifiers, filters, power rails, insulated rail brackets, and surge arresters were also considered. The system was modelled and simulated in the electromagnetic specialist software named Electromagnetic Transients Program Restructured Version (EMTP-RV). The EMTP-RV is a software suited for the transient study specifically in handling an electromagnetic time constant which is significantly smaller (faster transient), ranging in duration from microseconds to seconds [30]. It is considered as having the most advanced user-defined modelling capabilities and the most numerically stable time-domain software. In lieu of the unavailability of data related to the LRT Kelana Jaya line, the parameters considered in designing the user-defined devices modelled for this study were adopted as in [31] and as exemplified in Figure 3. 

## 3. Results and Discussion

**Figure 4:**
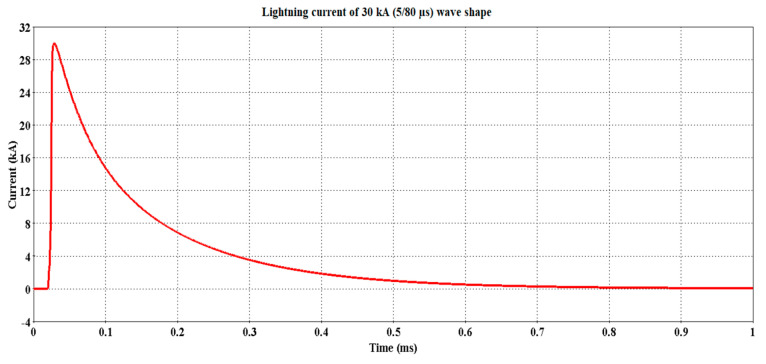
Lightning current 30 kA (5/80 µs) waveshape.

**Figure 5:**
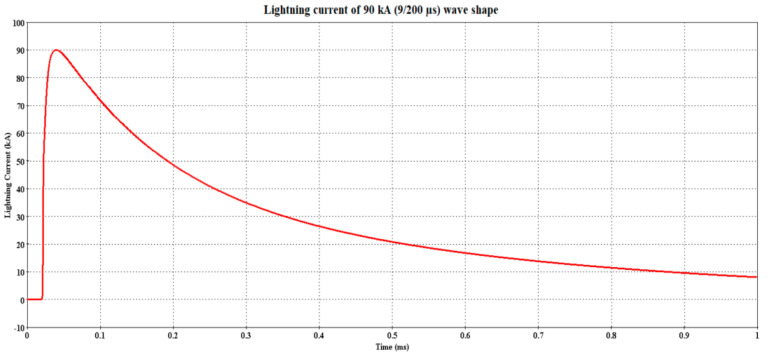
Lightning current 90 kA (9/200 µs) waveshape.

Due to the unavailability of the lightning current parameters for the LRT Kelana Jaya line, the parameters considered in computing the I_0_ followed the established values from the measurements of Berger et al. with probabilities of 50% and 5% occurrence, as shown in Table 1. The resultant lightning current based on the parameters in Table 1 is shown in Figure 4 and Figure 5, i.e., 30 kA (5/80 µs) and 90 kA (9/200 µs), respectively. 

As for the LRT Kelana Jaya line, the simulation model was based on the parameters depicted in Table 2. The justification of the selected Table 2 parameters is tabulated in Table 3.

**Figure 6:**
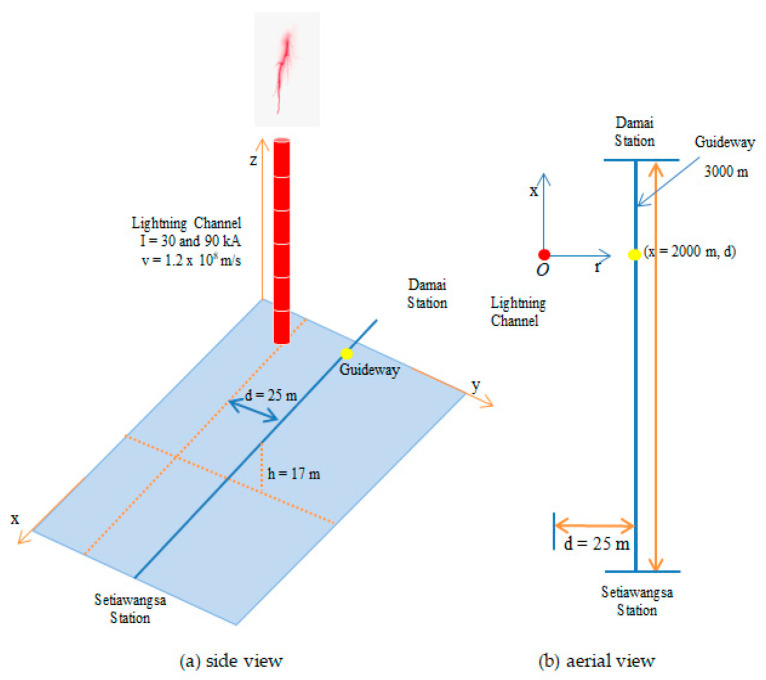
Illustration of the simulation configuration.

Referring to Figure 6, the distance is essentially limited to 2000 m as to highlight the impact of the induced effects on different bracket materials, namely the Cycloaliphatic Epoxy A (CEA) and the Glass Reinforced Plastic (GRP). The performances of these two materials were superimposed and compared with the Cast Epoxy (CE) bracket, which is currently being used in the LRT Kelana Jaya line. 

**Figure 7:**
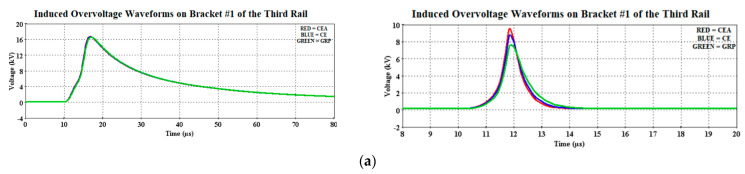

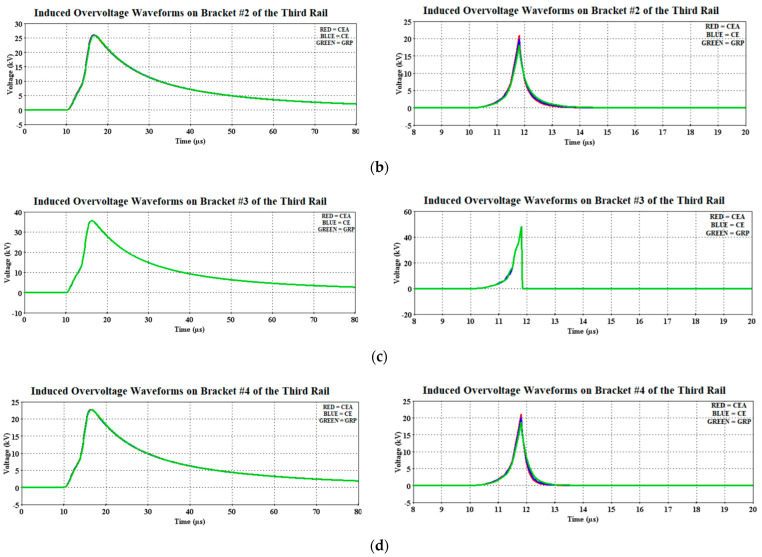
Induced overvoltage waveforms on the insulated rail brackets, i.e., (**a**) first bracket, (**b**) second bracket, (**c**) third bracket, and (**d**) fourth bracket, for both 30 kA (**left**) and 90 kA (**right**).

The induced overvoltage profiles for the CE, CEA, and GRP brackets are shown in Figure 7. There were only four brackets needed in the rail model portraying the 3 km distance from Setiawangsa Station to Damai Station, where one bracket is installed for every 1-km distance.

**Figure 8:**
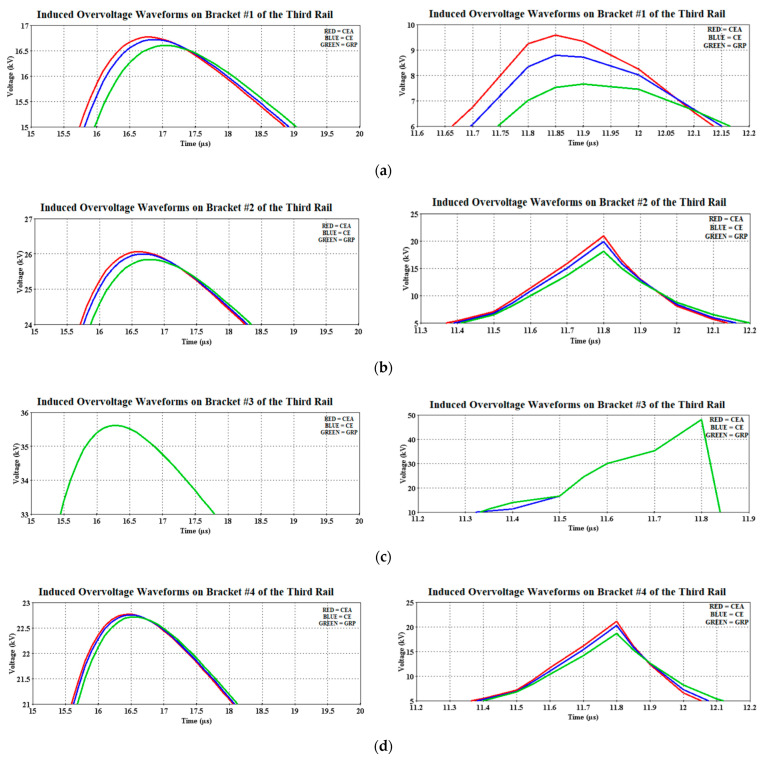
Magnified Figure 7 for a clearer view. (**a**) First bracket, (**b**) second bracket, (**c**) third bracket, and (**d**) fourth bracket for both 30 kA (**left**) and 90 kA (**right**).

**Figure 9:**
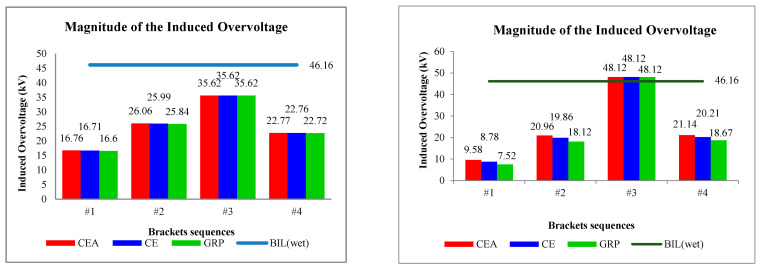
Induced overvoltage magnitude on the insulated rail brackets for both 30 kA (**left**) and 90 kA (**right**).

It was observed that the induced voltages on the brackets due to 30 kA were not much different from one another as compared with the ones caused by the 90 kA, in particular on the third bracket (Bracket #3). Figure 8 shows the magnified peak induced overvoltage, with the solid red, blue, and green lines representing the CEA, CE, and GRP materials, respectively. Whilst Figure 9 shows the corresponding magnitudes obtained based on Figure 8. Note that the solid green waveform in Figure 7 and Figure 8c represents the superimposed waveforms for other materials and this applies to all the solid green waveform results obtained in this study.

Since the impact of transient induced overvoltage has no influence on the performance of the fourth rail, further analysis of the fourth rail will not be discussed in this paper. The credible explanation that can be inferred is that the return stroke current model and the model function parameters as tabulated in Table 1 are the representation of a negative first return stroke current, hence explaining the dismissive reaction of the fourth rail towards the induced overvoltage.

Figure 8 shows the distinction between the bracket material performances when imposed with 30 kA (5/80 µs) and 90 kA (9/200 µs) waveforms, where different magnitudes of the induced overvoltage were observed. Note that the CE bracket was made as the reference case when comparing its performance with the CEA and GRP brackets. For example, all the three materials show the same magnitude of induced overvoltages recorded at the third bracket, due to their close distances to the lightning strike location and consequently experienced the highest induced overvoltage of 35.62 kV, before parting ways either bound to the Setiawangsa Station or to the Damai Station. This was then followed by the magnitudes recorded at the second, fourth, and first bracket. This description applied to both the 30 and 90 kA cases.

Overall, when comparing the induced magnitude of CE from the 30 kA case, the GRP was recorded to have a lower induced magnitude, while the CEA recorded a higher magnitude, taken at the same brackets as shown in Figure 9, indicating the difference of 0.58% for the second bracket, 0.18% for the fourth bracket, and 0.66% for the first bracket. On the other hand, the percentage increase of induced overvoltage due to the usage of CEA material as the insulated rail bracket were 0.27%, 0.04%, and 0.3%, for the second, fourth, and first CE brackets, respectively. It would be safe to say that the recorded induced magnitudes are lower than the BIL of 46.16 kV, except for the case of 90 kA for bracket #3, which exceeds the BIL. The GRP material was found to perform slightly better in the case of 90 kA with almost 1–2 kV voltage differences compared to CE and CEA.

On another perspective, GRP has the highest reduction with 53.4%, followed by CE with 53.09% and CEA at 52.95% when comparing with the BIL in the case of 30 kA. Whilst for the 90 kA case, the GRP bracket shows the highest reduction with 84.37%, followed by CE with 81.75% and CEA at 80.09%. This is acknowledged by the fact that GRP has the highest permittivity constant among these three materials and thus, provides an excellent electric stress controller, which is capable of reducing the stress to an acceptable desired value [35,36]. 

**Figure 10:**
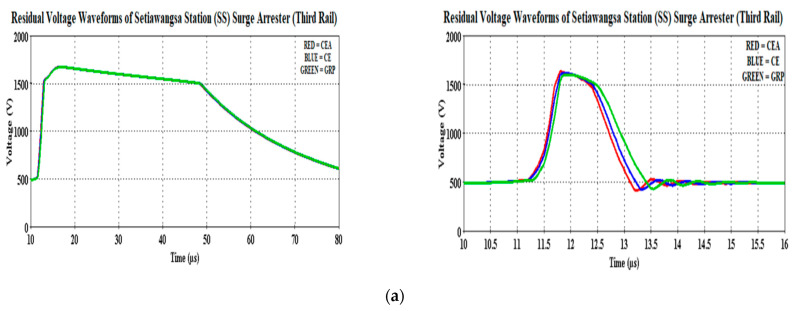

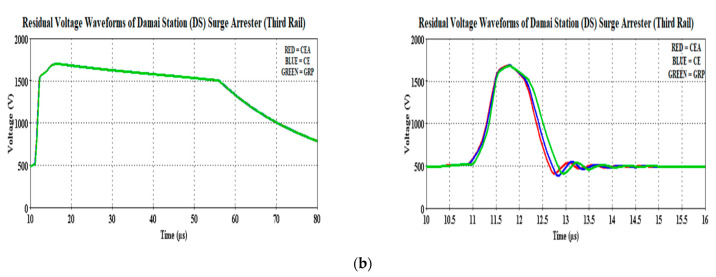
Residual voltage waveforms of the (**a**) Setiawangsa Station and (**b**) Damai Station surge arresters for both 30 kA (**left**) and 90 kA (**right**).

Figure 10 shows the residual voltages recorded by the station surge arresters for both cases. It was observed that the waveshapes of the arrester residual voltages were not much different from the reference case. As described in Figure 6, the lightning origin location was much closer to the Damai Station and thus, explained the higher clamped voltage magnitude at that station arrester (Figure 10b). Nonetheless, the high voltage current managed to be redirected to the ground within 40.1 µs, diminishing nearly 11% from the initial voltage magnitude as analyzed through Figure 10b for the 30 kA case. To be precise, the GRP bracket was able to redirect its high voltage current into 40.2 µs, diminishing 11.06% from its initial voltage. Similarly for the case of the CEA bracket where the same time lapse as per the GRP was obtained but only to diminish 0.05% more of the residual voltage than the reference case. However, in the case of 90 kA, the maximum peak has reached within 11.8 µs, regardless of the bracket-type materials, before receding to a lower magnitude of 0.3 µs later. 

**Figure 11:**
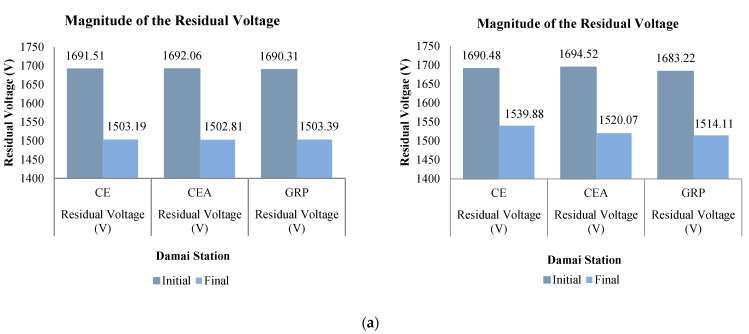

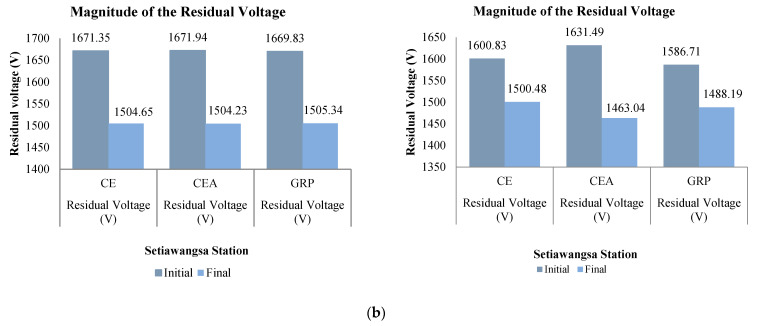
Residual voltage magnitudes of the (**a**) Setiawangsa Station and (**b**) Damai Station surge arresters for both 30 kA (**left**) and 90 kA (**right**).

Referring to Figure 11b, the GRP bracket recorded the lowest initial magnitude at 1683.22 V, which is 0.43% lower from the CE bracket. The arrester also managed to diminish 10% of its initial residual voltage to the ground within 0.3 µs, which is similar to the CEA bracket. Having the station surge arrester installed at the Setiawangsa Station has helped ease the high energy caused by the 30 and 90 kA lightning surge current, which can be seen in Figure 10a.

Although there is no significant difference recorded for the case of 30 kA, a slightly improved performance can be observed in the case of 90 kA. The reduction of the initial residual voltages between the two stations was 5.3% for the CE bracket, with the most reduction obtained for the GRP bracket at 5.73%. Figure 11a shows the induced overvoltage clamped by the arrester at a lower magnitude, which signified the quick speed-of-response of the arrester as opposed to when the arrester clamped a little later, which would make the arrester clamp at a higher magnitude and thus, jeopardize the downstream equipment [37]. 

**Figure 12:**
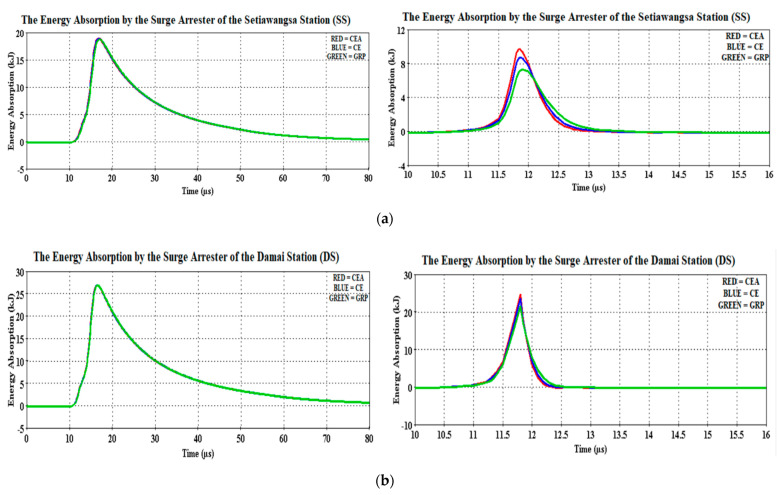
Energy absorption waveforms of the (**a**) Setiawangsa Station and (**b**) Damai Station surge arresters for both 30 kA (**left**) and 90 kA (**right**).

**Figure 13:**
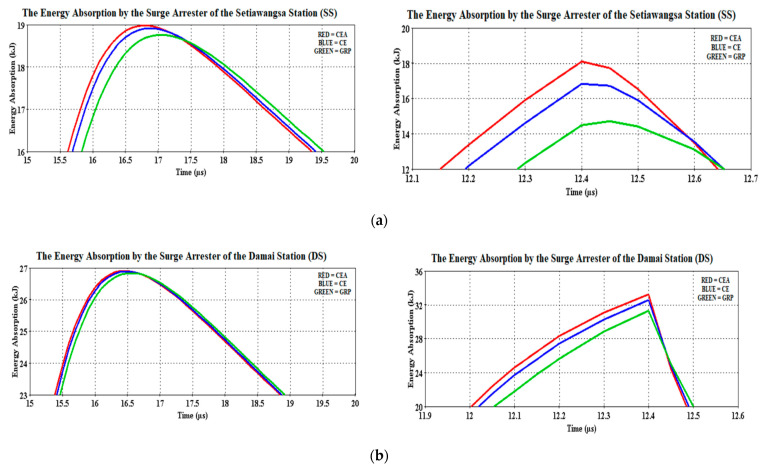
Magnified version of Figure 10. (**a**) Setiawangsa Station and (**b**) Damai Station surge arresters for both 30 kA (**left**) and 90 kA (**right**).

**Figure 14:**
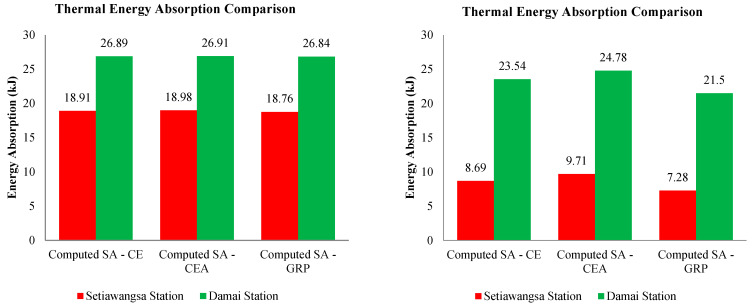
Comparison of the arrester thermal energy absorption between the computed bracket material cases for both 30 kA (**left**) and 90 kA (**right**).

Although no significant differences were observed for the case of 30 kA, the GRP bracket has recorded a lower clamping voltage and therefore it is reasonable to say that its thermal energy absorption was lower among the three materials, as discussed earlier and shown in Figure 12 and Figure 13. Having said that, it is not to judge the arrester performance based on the low thermal energy, instead it is just to show that a comparison was made between the computed energy absorption and the arrester’s own thermal energy absorption limit. An excellent example was in the case of 90 kA, depicted in Figure 12 (with the magnified version as in Figure 13) where for the Setiawangsa Station arrester, the GRP bracket contributed the lowest energy absorption of 7.28 kJ, followed by CE with 8.67 kJ and CEA at 9.71 kJ. Whilst for the Damai Station arrester, the GRP bracket recorded the lowest energy absorption, with 115% more than the limit allowed, i.e., 10 kJ, compared to 135.4% from the CE bracket and 147.8% from the CEA bracket, as shown in Figure 14. 

Should the energy handling capacity of the line arrester be exceeded, this would eventually lead to an internal arc and equipment breakdown. In the case of 90 kA, the high energies were dissipated within less than 1 µs and if they were able to remain cool against the system’s continuous operating voltage, then it is said to be able to thermally withstand the high energy.

Thus, by referring to Figure 14, it can be understood that the arrester of the Damai Station absorbed more energy as compared to the arrester at the Setiawangsa Station, considering the lightning strike point is much closer to the Damai Station. The GRP bracket was found to coordinate better with the station arrester, where it absorbed more than the limit permitted, i.e., 10 kJ, with 87.6% more at the Setiawangsa Station and 168.4% more at the Damai Station for the case of 30 kA. 

The better energy absorption of GRP as compared to others has provided a better margin of protection for the 12-pulse rectifier circuit. The margin of protection of the rectifier was calculated by the expression of U_pl_, 10 kA, 8/20 µs < BIL (device to be protected)/1.4, considering the BIL of the 12-pulse rectifier is 3600 V [38]. This has resulted in 2.5 kV, which puts the arrester residual voltage barely less than the margin of the protection voltage. Rest assured, the arresters may still work reliably and safely even though the energy absorption exceeded their standard allowable limit, given that the arresters have the time to cool down. The necessary cool-down time for the arresters depends on their construction, the ambient temperature, and the applied voltage. However, normally the cool-down time typically lies between 45 and 60 min [39]. It is also worth noting that the changes and variations in results could be significant when a higher magnitude of lightning stroke (with different waveshapes) is injected on the system, and in this case, it was the 90 kA (9/200 µs). 

In short, all these analyses with 30 kA (5/80 µs) and 90 kA (9/200 µs) lightning current on the LRT Kelana Jaya line have demonstrated the effect of insulation coordination between the CE, CEA, and GRP brackets with the station arrester.

## 4. Conclusions

This study has provided a useful insight obtained through the simulation carried out on the performances of the insulated rail brackets and the station surge arresters subjected to an indirect lightning stroke nearby. The lightning current was modelled by the sum of two Heidler functions representing the current at the channel base and the lightning induced overvoltage was modelled using the Rusck equation.

It is perhaps premature to conclude which type of rail bracket is superior when only a single stroke of 30 kA (5/80 µs) is considered. As such, results from the 90 kA (9/200 µs) were also included to support and highlight the influence of the bracket material. The preliminary analyses came to a conclusion that the escalation of the magnitude of the lightning induced overvoltage on the brackets was influenced by the bracket materials, which depends on the properties of the material itself, i.e., permittivity. A material with better permittivity such as the GRP material is known to be an excellent electric stress controller and hence, capable of reducing the stress to an acceptable desired value. Therefore, it results in better insulation coordination with the station surge arrester in terms of residual voltage and energy absorption capacity of the station arrester.

In short, these simulations were carried out to ensure the reliable operation of the fourth rail transit. Considering that Malaysia is known for having one of the highest lightning flash densities in the world, this work is considered to be critical to the railway operator and owner. Moreover, it is worth noting that these findings were based on the initial lightning current injected onto the system, i.e., 30 kA (5/80 µs) and 90 kA (9/200 µs) and therefore, the results would likely be different should other lightning currents with different magnitudes and waveshapes be considered.

## Figures and Tables

**Table 1:** Parameters of Heidler Function for I_0_ Peak.

Occurrence	Heidler Function Setting				I_0_ (kA)
%	I_1_ (kA)	τ_11_ (µs)	τ_21_ (µs)	n_1_	I_0_
50 [32]	9.075	3.42	40.24	2	30 (5/80 µs)
	I_2_ (kA)	τ_12_ (µs)	τ_22_ (µs)	n_2_	
	20.515	4.793	153.46	10	
	I_1_ (kA)	τ_11_ (µs)	τ_21_ (µs)	n_1_	
5 [32]	46.49	5.86	143.997	2	90 (9/200 µs)
	I_2_ (kA)	τ_12_ (µs)	τ_22_ (µs)	n_2_	
	41.548	41.759	592.86	10	

**Table 2:** The relative simulation variations.

Operating System Voltage (V)	System Length (m)	Height (m)	Insulated Rail Bracket	Arrester(Residual Voltage (kV))	I_0_ (kA)	Velocity (v) (10^8^ m/s)	Strike Point	
							Horizontal(d) (m)	Vertical (x) (m)
750	3000	17	CE	3EB4-010 (2.4)	30 and 90	1.2	25	2000
			CEA					
			GRP					

**Table 3:** Justification of the parameters determination.

Parameter		Value	Justification
Guideway Height (m)		17	The proposed height follows the equation of span-to-height ratio of 3 as to create an aesthetical appearance. Furthermore, a higher elevation creates a more open and lighter area underneath the guideway [33].
Lightning	Current Peak (kA)	30 and 90	The selected magnitude is the typical magnitude of the negative first return stroke with 50% and 5% occurrences worldwide. The replication of the magnitude and its waveshape follows the assumption made towards the Heidler function parameters in the IEC 62305-1 [32].
	Velocity (×10^8^ m/s)	1.2	The selected velocity is the commonly accepted value as it lies in between c/3 and 2c/3, the typical velocities range of a return stroke lightning current, with c as the speed of light in free space; 2.99792458 × 10^8^ m/s [26,34].
Strike Distance	Horizontal (m)	25	The short distance is considered based on the fact that the LRT Kelana Jaya line runs through the heart of Greater Kuala Lumpur, a city that housed the majority of the Malaysia government administration buildings, businesses, entertainments, and leisure landmarks where void spaces between the guideway and the buildings are limited. In addition, 25 m is within the range of Protection Zone Law [34]; 2.5 to 80 m.
	Vertical (m)	2000	The distances were selected for assessing the impact of induced overvoltages on the insulated rail bracket (2000 m).
Bracket Material		CE, CEA, GRP	The rail bracket is made from polymer-based materials, i.e., CE, CEA, and GRP which have a different permittivity value.
Surge Arrester		3EB4-010 10 kA at 8/20 µs and 2.4 kV	The selected surge arrester was based on its rated voltage (1.0 kV), MCOV (1.0 kV), and most importantly based on its residual voltage (2.4 kV) at a nominal discharge current (10 kA at 8/20 µs), the arrester energy limit is 10 kJ.

## Data Availability

Not applicable.
